# Autophagy Controls an Intrinsic Host Defense to Bacteria by Promoting Epithelial Cell Survival: A Murine Model

**DOI:** 10.1371/journal.pone.0081095

**Published:** 2013-11-19

**Authors:** Sun-Young Chang, Se-Na Lee, Jin-Young Yang, Dong Wook Kim, Joo-Heon Yoon, Hyun-Jeong Ko, Michinaga Ogawa, Chihiro Sasakawa, Mi-Na Kweon

**Affiliations:** 1 Mucosal Immunology Section, Laboratory Science Division, International Vaccine Institute, Seoul, Korea; 2 Laboratory of Microbiology, College of Pharmacy, Ajou University, Suwon, Kyeonggi-do, Korea; 3 College of Pharmacy, Hanyang University, Ansan, Kyeonggi-do, Korea; 4 Department of Otorhinolaryngology, Yonsei University College of Medicine, Seodaemun-Gu, Seoul, Korea; 5 Laboratory of Microbiology and Immunology, College of Pharmacy, Kangwon National University, Chuncheon, Kangwon-do, Korea; 6 Department of Bacteriology I, National Institute of Infectious Diseases, Tokyo, Japan; 7 Nippon Institute for Biological Science, Tokyo, Japan; 8 Medical Mycology Research Center, Chiba University, Chiba, Japan; Charité-University Medicine Berlin, Germany

## Abstract

Cell death is a critical host response to regulate the fate of bacterial infections, innate immune responses, and ultimately, disease outcome. *Shigella* *spp.* invade and colonize gut epithelium in human and nonhuman primates but adult mice are naturally resistant to intra-gastric *Shigella* infection. In this study, however, we found *Shigella* could invade the terminal ileum of the mouse small intestine by 1 hour after infection and be rapidly cleared within 24 h. These early phase events occurred shortly after oral infection resulting in epithelial shedding, degranulation of Paneth cells, and cell death in the intestine. During this process, autophagy proceeded without any signs of inflammation. In contrast, blocking autophagy in epithelial cells enhanced host cell death, leading to tissue destruction and to inflammation, suggesting that autophagic flow relieves cellular stress associated with host cell death and inflammation. Herein we propose a new concept of “epithelial barrier turnover” as a general intrinsic host defense mechanism that increases survival of host cells and inhibits inflammation against enteric bacterial infections, which is regulated by autophagy.

## Introduction

Host cell death is an intrinsic immune defense strategy in response to microbial invasion [1]. “The demise of infected cells” plays a pivotal role in sacrificing damaged cells, eliminating pathogens, limiting microbial replication, and emitting alarm signals. Previous studies demonstrated that *Shigella* invasion induces both necrosis-like death of epithelial cells and apoptosis-like death of macrophages in a caspase-1-independent manner [2,3]. In contrast, bacterial pathogens deploy multiple mechanisms to postpone host cell death that favor infection. *Shigella* organisms prevent host cell death by NF-κB activation until it has successfully replicated and spread into the surrounding epithelia [4]. 

While *Shigella* species cause bacillary dysentery by invading colon epithelium and promoting a strong inflammatory response in human and nonhuman primates, adult mice are naturally resistant to intra-gastric *Shigella* infection [5]. We and others have shown that newborn mice (4–7 days after birth) are transiently susceptible to *Shigella* infection and intriguingly that the maturation status of Paneth cells and secretion levels of antimicrobial peptides in the mouse gut appear to correlate with their susceptibility to *Shigella* infection [5–7]. However, the exact mechanism for the natural resistance of adult mice to intra-gastric *Shigella* infection is unknown. 

Autophagy is a tightly regulated process for the degradation of a cell's own components through the lysosomal machinery involving cell growth, development, and homeostasis [8]. Autophagy is also regarded as one of the innate immunity effectors against intracellular bacterial infection [9]. For instance, *Streptococcus pyogenes* (Group A *Streptococcus*; GAS)-specific autophagy appears to selectively sequester and eliminate bacteria, which is distinct from nonselective canonical autophagy [10]. When the innate defense system recognizes invasive bacterial pathogens and their infection processes, autophagic proteins act as cytosolic sensors to rapidly launch the autophagic pathway [11]. However, many intracellular bacterial pathogens deploy highly evolved mechanisms to evade autophagic recognition, manipulate the autophagic pathway, and remodel the autophagosomal compartment for their own benefit [12]. *Shigella* *spp.* have been known to escape autophagy by secreting IcsB to enable binding to VirG, thus preventing autophagy by limiting VirG’s interactions with the host’s autophagy protein, Atg5 [13]. 

Here we examine the acute host response in adult mice against intra-gastric *Shigella* infection. Unexpectedly, host cells in the terminal ileum region of the small intestine were found to undergo a bold death process induced by microbes. Although oral *Shigella* infections never induce inflammatory signals, the autophagic process induced by infection may relieve the cellular stress to inhibit inflammation. 

## Materials and Methods

### Mice and Bacteria strains

C57BL/6 (B6) mice were purchased from Charles River Laboratories (Orient Bio Inc., Sungnam, Korea). LC3-GFP knock-in mice, ATG5 ^flox/flox^ mice, and ATG7 ^flox/flox^ mice were purchased from RIKEN BRC (Tsukuba-shi, Ibaraki, Japan). Villin-Cre mice were purchased from Jackson Laboratory (Bar Harbor, ME). All mice were maintained under specific pathogen-free conditions in the experimental facility at the International Vaccine Institute (Seoul, Korea) where they received sterilized food and water *ad libitum*. This study was carried out in strict accordance with the recommendations in the Guide for the Care and Use of Laboratory Animals of Ministry of Food and Drug Saftey. The protocol was approved by the Committee on the Ethics of Animal Experiments of the International Vaccine Institute (IACUC PN2011-002). Virulent *Shigella flexneri 5a* (M90T), IpaB4 deletion mutant (M90T∆IpaB4), enteropathogenic *Escherichia coli* (EPEC) from Dr. Chihiro Sasakawa (University of Tokyo, Japan), and *Citrobacter rodentium* from ATCC (Rockville, MD) were used for infection. Enterohemorrhagic *E. coli* (EHEC, O157:H7) and verotoxin-deficient O157:H7 (O157:H7∆verotoxin) were provided by the Korea Center for Disease Control and Prevention (Chungwon, Chungcheongbuk-do, Korea). For bacterial infection, each mouse was orally administered 5 x 10^9^ bacteria.

### Bacteria count (CFU)

To assess the numbers of bacteria from intestinal tissues of non-infected and *Shigella*-infected mice, tissues were extensively washed in PBS with gentamicin (50 μg/ml) to remove luminal and simply attached bacteria. Tissues were homogenized and plated onto TSB agar plates containing streptomycin since M90T strain is streptomycin resistant. After overnight culture at 37°C, colonies were counted. To assess bacterial shedding, feces were suspended at 100 mg/100 µl of PBS, diluted, and plated onto TSB agar plates containing streptomycin.

### Histology

Distal regions of mouse ileum were washed with PBS containing gentamicin and fixed in 4% formaldehyde for 1 h at 4°C. The tissues were dehydrated by gradually soaking them in alcohol and xylene and then embedded in paraffin. The paraffin-embedded specimens were cut into 5-µm sections, stained with hematoxylin-eosin (H&E), and viewed with a digital light microscope (Olympus, Tokyo, Japan). TUNEL (Roche, Mannheim, Germany), E-cadherin (BD Pharmingen, San Diego, CA), and CD11b-PE/Gr1-FITC (BD Pharmingen) were stained using cryo-sections or paraffin sections, according to each manufacturer’s instructions. To detect green fluorescent protein (GFP)-expressing bacteria in the intestinal tissue or LC3 puncta from LC3-GFP knock-in mice, the frozen sections of ileal tissues were prepared and viewed under a confocal scanning laser microscope (Carl Zeiss, Göttingen, Germany). We evaluated autophagy by assessing single cells with more than four LC3 GFP puncta in the cytosol as shown on confocal images.

### Disease score

The levels of tissue destruction were addressed using H&E-stained samples of ileum after bacterial infection. The category and parameters including host cell death (0–3/each category, crypt, epithelium, lamina propria [LP], and muscles), epithelium shedding (0–4), barrier integrity (0–4), inflammation (0–4), and goblet cell hyperplasia (0–4) are described in Figure S1.

### Real time PCR and Gene Chip

For real time polymerase chain reaction (PCR) analysis, total RNA was extracted using an RNeasy kit (Qiagen, Hilden, Germany) and cDNA was synthesized by Superscript II reverse transcriptase with oligo(dT) primer (Invitrogen, Carlsbad, CA). Specific primer sets are listed in Table S1. Then, gene expression quantification was performed using an ABI PRISM sequence detection system (Applied Biosystems, Foster, CA) in which the levels of mRNA expression are displayed as the expression units of each target gene relative to the expression units of GAPDH. For gene chip analysis, total RNA was amplified and purified using the Ambion Illumina RNA amplification kit (Ambion, Austin, TX) to yield biotinylated cRNA according to the manufacturer’s instructions. In total, 750 ng of labeled cRNA samples were hybridized to MouseRef-8 V2.0 expression bead array for 16–18 h at 58°C, according to the manufacturer's instructions (Illumina, San Diego, CA). Detection of array signal was carried out using Amersham fluorolink streptavidin-Cy3 (GE Healthcare Bio-Sciences, Little Chalfont, UK) as described in the bead array manual. Arrays were scanned with an Illumina bead array reader confocal scanner according to the manufacturer's instructions. Array data export processing and analysis were performed using Illumina BeadStudio v3.1.3 (Gene Expression Module v3.3.8). 

### Electron microscopy

For scanning electron microscopy (SEM), intestinal tissues of distal ileum were fixed in 2% glutaraldehyde and 2% paraformaldehyde in 0.1 M sodium cacodylate for 1 h at room temperature. After being washed with PBS, specimens were treated with 1% osmium tetroxide for 1 h at room temperature and then dehydrated in graded ethanol solution. Dehydrated tissues were critical point dried with CO_2_, sputter coated, and observed with a low vacuum-SEM (S-3500N; Hitachi Sciences Systems, Santa Clara, CA). For transmission electron microscopy (TEM), tissue samples were sectioned and viewed with an energy filtering TEM unit (LEO-192AB OMEGA, Carl Zeiss).

### Cytokine from intestinal homogenate

Distal ileum tissue samples were weighed (200 mg/ml) and homogenized with Tris-EDTA buffer (10 mM Tris-HCl and 1 mM EDTA, pH 7.4, 0.05% sodium azide, 1% Tween-80, protease inhibitor cocktail), centrifuged at 11,000 x g for 10 min at 4°C, and supernatant was collected. Cytokine from tissue homogenates was measured using the cytometric bead array-mouse inflammation kit (BD Pharmingen) according to the manufacturer’s instruction.

### Statistics

GraphPad Prism software (GraphPad Software, La Jolla, CA) was used for statistical analysis. Student’s *t* test or ANOVA were used for comparisons. All results are expressed as mean ± SD.

## Results

### *Shigella* invasion of intestinal tissue is followed by rapid clearance by host defense

**Figure 1:**
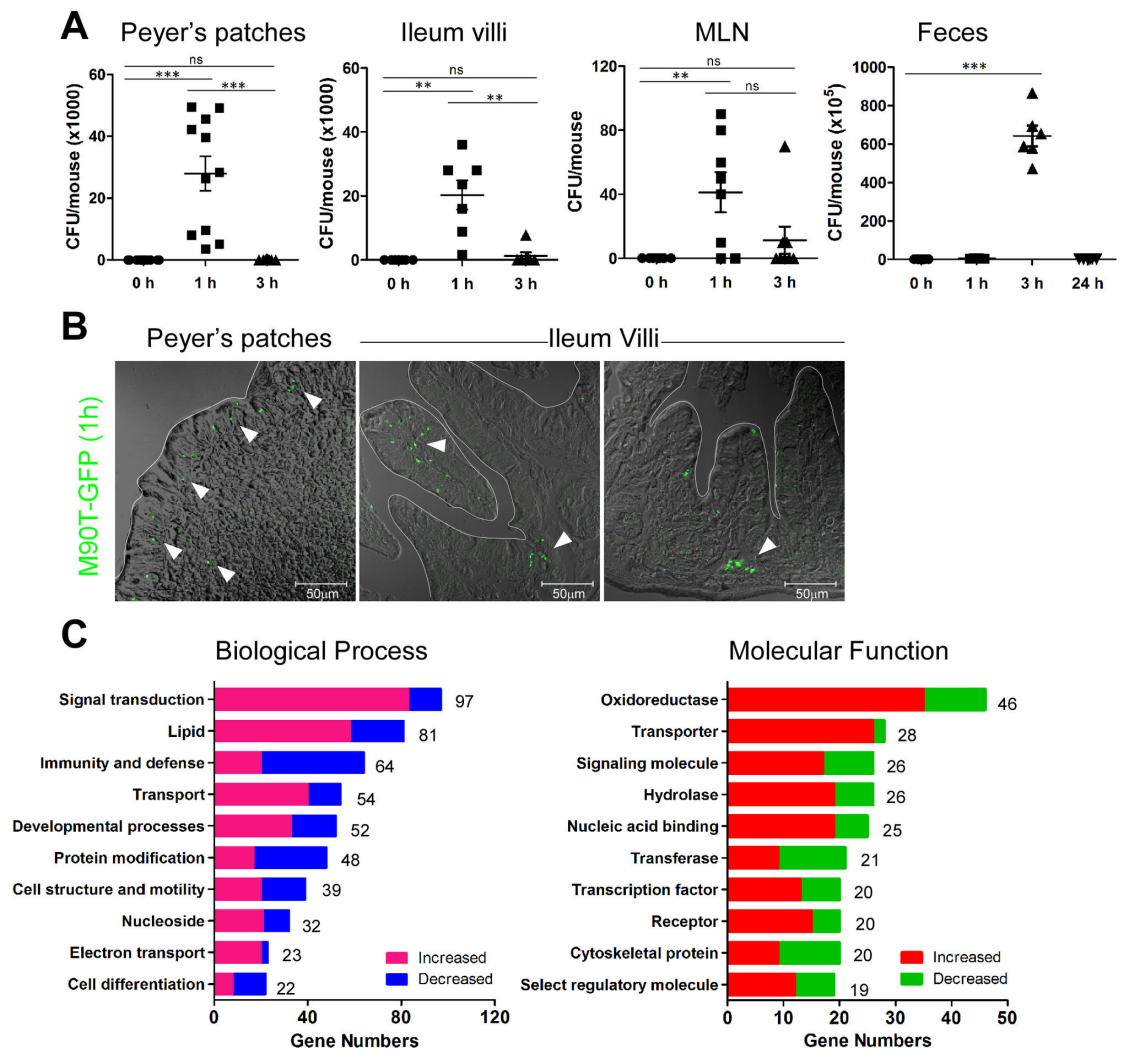
*Shigella* *spp.* invade mouse intestine and change host biological processes. (A) Colony-forming units (CFU) of M90T from Peyer’s patches (PP), ileum, mesenteric lymph node (MLN), and feces by time after oral M90T infection. (n >10) Not significant (ns), **< P=0.001, ***< P=0.0001. (B) Green fluorescent protein expressing M90T (arrowhead) in the PP and villi of ileum 1 h following oral M90T infection. (n=8) (C) Increased and decreased biological function analyzed from gene expression profile of terminal ileum tissue 1 h following oral M90T infection compared to uninfected tissue.

To investigate the natural resistance of adult mice to enteric pathogens, 6~8-week-old B6 mice were challenged orally with virulent *S. flexneri* 5a (M90T; 5x10^9^) and assessed for patho-physiological changes of the gut at an early time point beginning 1 h after infection. We first determined whether oral *Shigella* *spp.* invade and colonize the murine intestine since it has been long believed that they cannot survive in murine intestine. After adult B6 mice were orally administered strain M90T, colony-forming units (CFU) were counted in homogenates of whole intestine tissues and feces. Unexpectedly, we detected considerable numbers of *Shigella* colonies in Peyer’s patches (PPs), terminal ileum villi, and mesenteric lymph node (MLN) 1 h after oral M90T infection (Figure 1A). Primary colonization sites were distal rather than the proximal and middle regions of the small intestines (Figure S2A). *Shigella* organisms disappeared within 3 h in the small intestine and within 1 day after infection in fecal CFU (Figure 1A). These findings occurred in both BALB/c and B6 background mice (data not shown). In order to address the localization of invasive shigellae, we traced M90T-expressing GFP following oral infection (Figure 1B). At 1 h following infection, M90T were detected even deep in the crypt region as well as in epithelial cells of PP and ileum villi. *Shigella* *spp.* induce inflammation and bloody diarrhea in human colon but not in small intestines [14]; however, in mice, oral *Shigella* infection did not target the colon (data not shown). Oral infection with M90T induced significant body weight loss beginning at day 1 in a dose-dependent manner, but the mice quickly recovered (Figure S2B). Regardless of weight loss, tissue damage was evident even after low infectious doses (Figure S2C). B6 mice orally administered the same dose of avirulent *S. flexneri 5a* (BS176) strain did not lose body weight (Figure S2D) and had low CFU titers in the PP and LP (data not shown). To address the role of type III secretion systems (T3SS), we infected mice orally with two kinds of T3SS-deleted *S. flexneri 5a* mutants (M90T∆*IpaD* and M90T∆*IpaB4*). Both T3SS mutants protected mice from body weight loss and did not damage tissue (Figure S2E and F). 

To address biological changes in host tissues, we analyzed gene expression profiles from the terminal ileum following oral M90T infection (Figure 1C, Figure S3, and Table S2). A number of genes were dynamically altered after oral M90T infection compared with samples from uninfected mice. This finding was confirmed by real-time PCR (Figure S3B and C). In particular, cytochrome p450 isoforms involved in drug metabolism (Cyp4v3, Cyp2c67), cation transporter (SLC22A4), and IL-33 were increased after *Shigella* infection (Table S2). These results suggest that *Shigella* organisms can infect the mouse intestine and then be rapidly cleared by active and dynamic host defense mechanisms rather than by passive expulsion.

### *Shigellae* induce tissue destruction at early stages of infection

**Figure 2:**
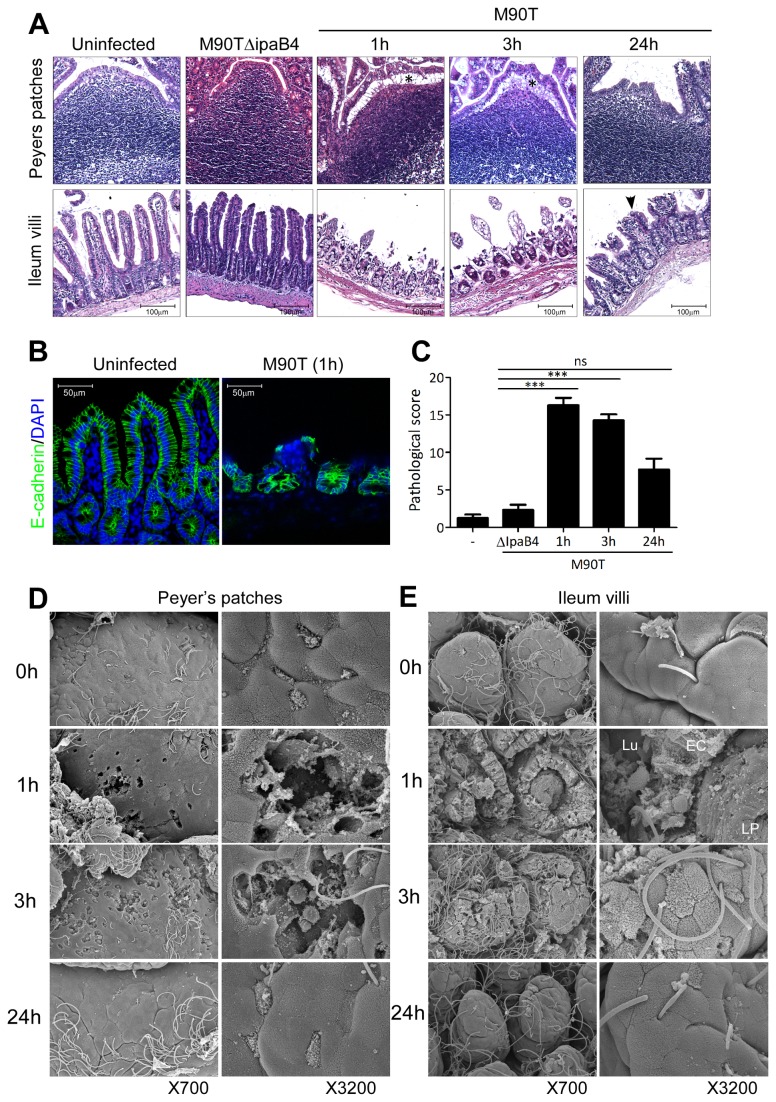
Tissue destruction of ileum by M90T infection followed by rapid recovery. (A) Hematoxylin-eosin (H&E) staining of terminal ileum following oral M90T or M90T∆IpaB4 infection. Epithelial shedding (*), tissue regeneration (arrowhead). (B) Confocal image of E-cadherin (green) for epithelium structure and DAPI (blue) staining for nucleus from terminal ileum. (C) Pathological score assessment from terminal ileum H&E histology following oral M90T infection by a blind test. (n >20). Not significant (ns), ***< P=0.0001. (D-E) Scanning electron microscopy (SEM) images of dome region of PP (D) and intestinal villi (E) in terminal ileum following oral M90T infection. Lumen (Lu), epithelial cells (EC), lamina propria (LP) (n=10).

To investigate the type of tissue remodeling following oral M90T infection, histopathological analysis revealed that at early time points (within several hours), epithelial sheets in the dome region of PPs of the terminal ileum were sloughing off from the basement membrane (Figure 2A). This phenomenon was most evident in the villi region where epithelial tissues were exfoliated and shed from the LP basement membrane (Figure 2A). E-cadherin staining clearly showed both shedding and damaged integrity of the epithelium following infection (Figure 2B). Although the cells in the LP, crypt, and epithelium were dying, the epithelium of the PPs and villi were rapidly regenerating and had renewed by 24 h after oral M90T infection. These unique pathological changes, which represent cell death (columnar epithelial cells, LP cells, crypt, and muscle) and epithelial shedding without inflammation, were scored as time points (Figure S1). Tissue disruption peaked at 1 h and was followed by tissue healing within 1 day (Figure 2C). Such tissue injury induced by M90T infection was dependent on T3SS since intragastric M90T∆ipaB4 infection did not produce any pathological changes (Figure 2A). 

Consistent with H&E histology, SEM showed single or multiple holes in the PP dome accompanied by damage to the LP with loss of the villous tips due to cell death and epithelial shedding (Figure 2D-E, Figures S4 and S5). Yet, these damaged tissues regenerated within 24 h of oral M90T infection.

Some *Shigella* strains such as *S. dysenteriae 1* produce Shiga toxin, while others including M90T do not [15]. To learn whether early tissue destruction is an M90T-specific response, we tested other *Shigella* strains (*S. dysenteriae 1, S. flexneri 2a* [YSH6000)]; Figure S6). *S. dysenteriae 1* and *S. flexneri 2a* induced similar pathological changes in the host gut (Figure S6A-B). To rule out the effect of excessive lipopolysaccharide, we examined murine ileum samples following oral gavage of lysates with 5 × 10^9^ CFU of M90T to mimic the administration of the equivalent dose of lipopolysaccharide. M90T lysates could not evoke host responses as with intact M90T (Figure S6C).

### *Shigella* infection induces host cell death in the intestine

**Figure 3:**
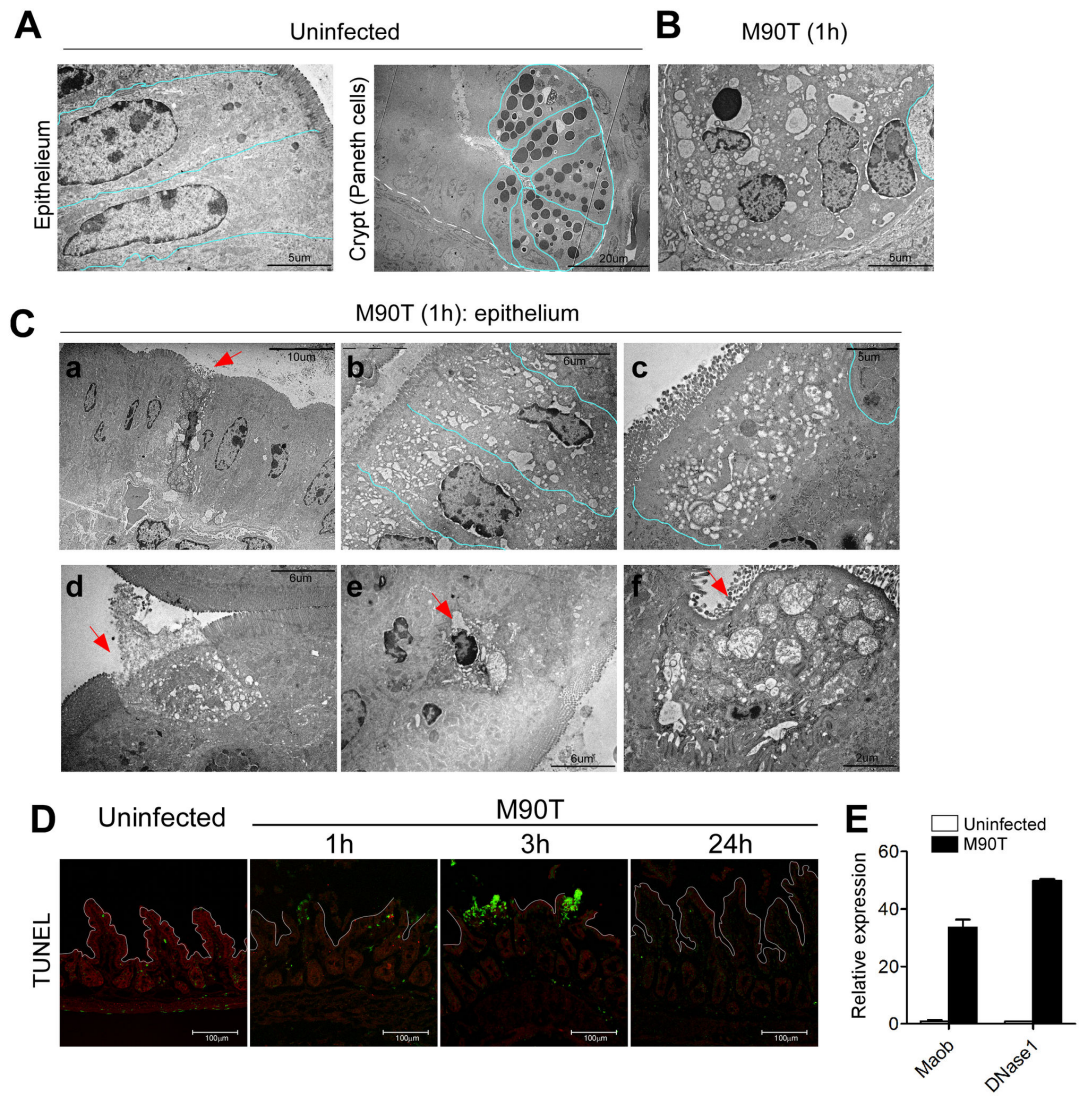
Intracellular alteration in Paneth cells of crypt and villous epithelial cells following oral M90T infection. Transmission electron microscopy (TEM) images of cellular level alterations in terminal ileum 1 h after oral M90T infection (n=10). (A) Uninfected villous epithelial cells and Paneth cells in the crypt show normal intracellular organelles. (B) In crypt Paneth cells, some cells have condensed nuclei and ruptured plasma membranes making floods of intracellular organelles. (C) TEM images of intestinal epithelial cells; a) single dying cells (arrow), b) adjacent multiple cells have swollen mitochondria and disorganized cytoskeletal elements, c) abundant vacuoles in cytoplasm. Pale blue lines denote individual cells, d-f) TEM images of cell death in villous epithelial cells. (D) TUNEL staining in terminal ileum following oral M90T infection (n=5). (C) mRNA expression of MaoB and DNase I from terminal ileum following oral M90T infection.

To further clarify host response at the cellular level, Paneth cells in the crypts and columnar epithelial cells in the villi were examined by TEM. The secretory granules, represented by unique black dots in the Paneth cells of the uninfected crypt, are crucial for secretion of anti-microbial peptides (Figure 3A). At 1 h after infection, these showed degranulation, irregular fusion, and excessive vacuolization (Figure 3B). Lysozyme expression and secretion were elevated after infection but secretory granules were totally collapsed in most of the ileal crypt epithelium (Figure S7). Cell death and the excess vacuolization could also be examined in the epithelial cells of villi (Figure 3C a-c). Some cells had swollen mitochondria and increased numbers of vacuoles representing autophagy [16]. These dramatic alterations inside cells were expected to induce death. Indeed, necrotic (Figure 3C-d) or apoptotic cell death (Figure 3C-e) and other complicated forms of cell death that showed swollen/ruptured mitochondria, irregular microvilli, and intact plasma membrane (Figure 3C-f) were found in the villi. The ratio of necrotic, apoptotic, and complicated forms of epithelial cell deaths evaluated from TEM images of the ileum 1 h after infection was 7:4:4. Interestingly, host cells in the LP, but not in the epithelium, were mainly TUNEL-positive, suggesting apoptotic cell death and peaked 3 h after oral infection (Figure 3D). MaoB, which is known to relate to apoptotic cell death in brain diseases [17], and DNase I, which is involved in necrotic cell death [18], were specifically increased following oral *Shigella* infection (Figure 3E). Isolated epithelial cells undergoing early (Annexin V^+^ propidium iodide [PI]^-^) and late apoptotic cells (Annexin V^+^PI^+^) were increased at 3 h after oral infection (Figure S8). Collectively, these findings show that host cells in the intestine following oral *Shigella* infection undergo differential cell death, mainly apoptosis in the LP versus necrosis in the epithelium. These data suggest that oral *Shigella* infection induces substantial host cell death in the mouse intestine.

### *Shigella* infection may enhance canonical autophagy in epithelial cells

**Figure 4:**
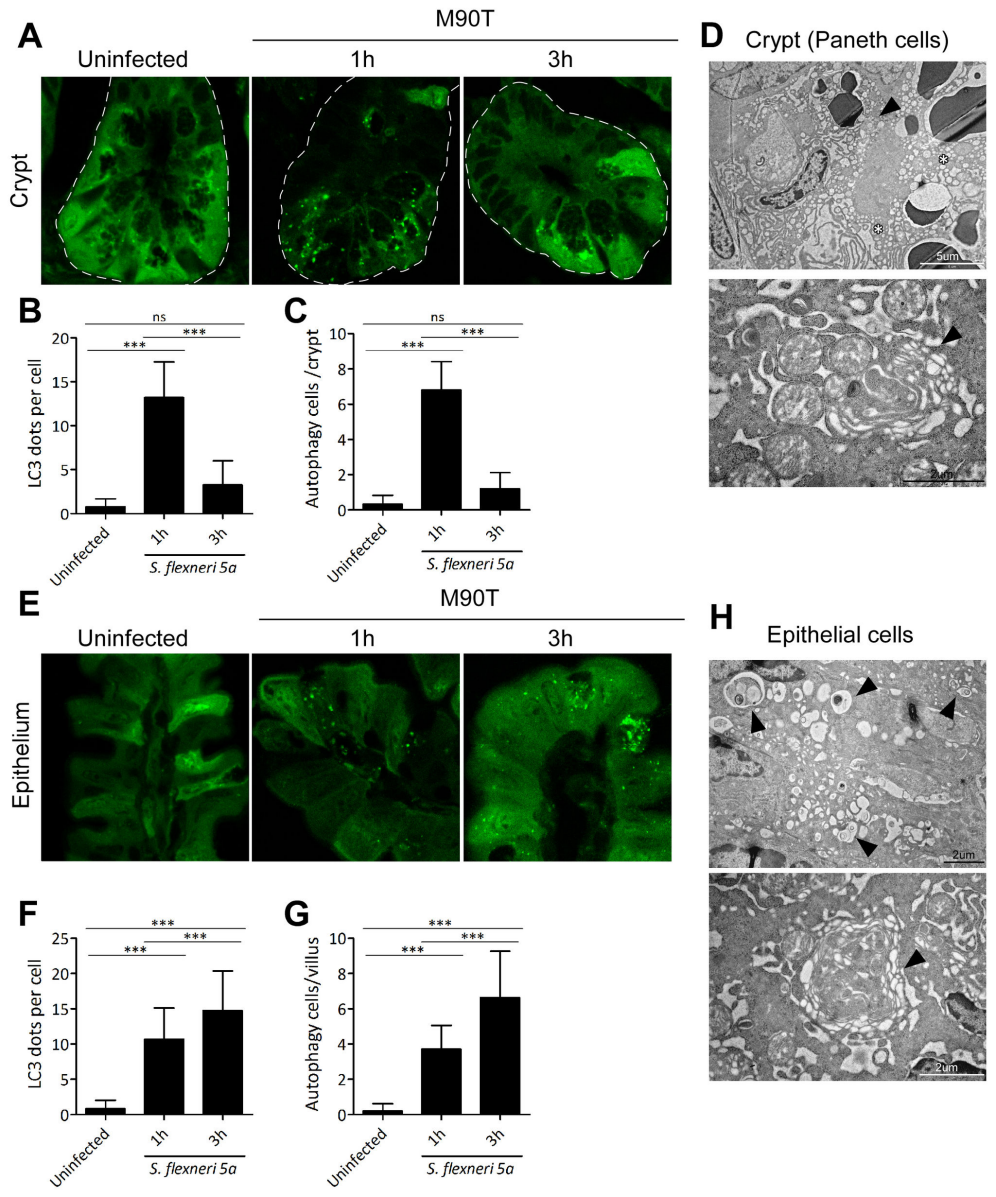
Oral *Shigella* infection-induced autophagy in Paneth and villous epithelial cells. After oral M90T infection, crypt (A-C) and villous epithelial cells (E-G) in the terminal ileum region of LC3 GFP transgenic mice were examined for autophagy induction as LC3-GFP puncta formation (n=10). Confocal images of GFP (LC3) puncta formation in the crypt (A) and villi (E). GFP puncta per cell and cells undergoing autophagy in crypt (B-C) and villi (F-G) were counted. TEM images of Paneth cells in the crypt (D) and epithelial cells in the villi (H) of wild type B6 mice 1 h following oral M90T infection. Representative data are from three independent experiments. Multi-layer autophagic vacuole (arrowhead) and excess vacuolization (star). Not significant (ns), ***< P=0.0001.

The morphological changes in the Paneth cells and excess vacuolization and swollen mitochondria in the epithelial cells (Figure 3) enabled us to see autophagy induction in depth. We examined the autophagic marker LC3 puncta in the crypt and epithelium of LC3-GFP transgenic mice following oral M90T infection. At 1 h after infection, LC3 puncta in the crypt (Paneth cells) were significantly increased compared to base levels, showing induction of autophagy. They returned to base levels within 3 h (Figure 4A-C). In the villi, LC3 puncta were also induced in both epithelial and goblet cells and continued to increase for up to 3 h (Figure 4E-G). Of note, double or multiple membrane structures, which are key features of autophagy, were found in both crypt (Figure 4D) and epithelial cells (Figure 4H). These process were considered canonical autophagy, not xenophagy, since some cells with LC3 puncta were not always co-localized with M90T (data not shown) and the LC3 puncta were not large enough to surround bacteria. These results suggest that M90T infection is capable of inducing autophagy in the intestine, which in turn could affect the regulation of inflammation.

### Autophagy by microbial infection may repress bacterial invasion, host cell death, and inflammation

**Figure 5:**
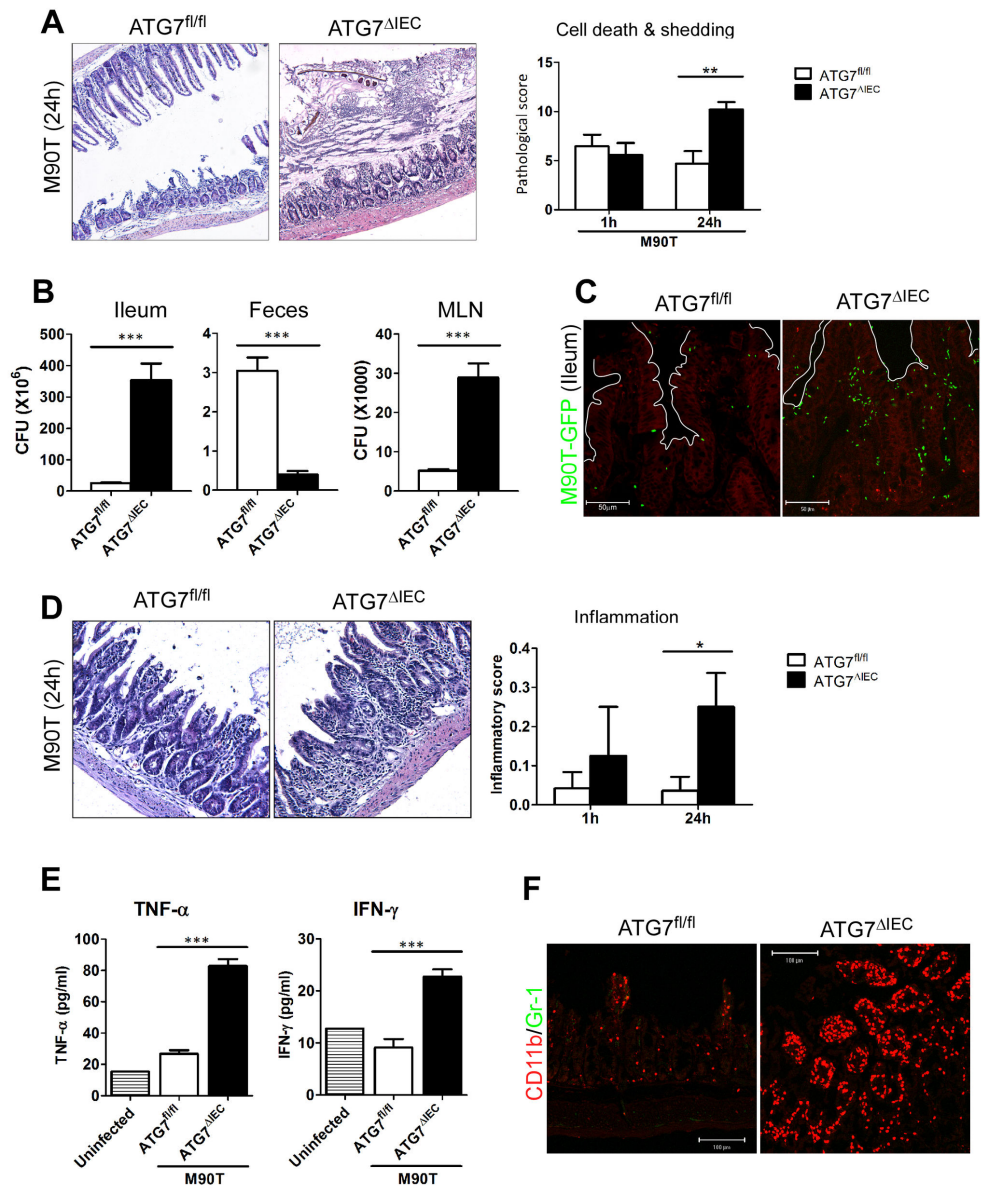
Autophagy process contributes to defense against bacterial invasion and regulation of pathogenic inflammation following enteric pathogen infection. After ATG7^fl/fl^ or ATG7^∆IEC^ mice were infected with oral M90T, terminal ileum was analyzed by time course (n=10). (A) Hematoxylin-eosin (H&E) staining of terminal ileum 24 h after infection. Representative images and pathologic score for cell death and shedding are shown. (B) CFU from ileum, mesenteric lymph nodes (MLN), and feces of ATG7^fl/fl^ or ATG7^∆IEC^ mice 1 h after infection. (C) Confocal image of green fluorescent protein (GFP)-expressing M90T that invaded the terminal ileum of ATG7^fl/fl^ or ATG7^∆IEC^ mice at 1 h after infection. (D) H&E staining of terminal ileum at 24 h after infection. Representative images and pathologic scores for inflammation were addressed. (E) TNF-α and IFN-γ from ileum tissue homogenates of ATG7^fl/fl^ or ATG7^∆IEC^ mice 24 h after infection. (F) Immuno-staining of terminal ileum of ATG7^fl/fl^ or ATG7^∆IEC^ mice 24 h after infection. CD11b^+^ monocyte-like cells (red), Gr-1^+^ neutrophil (green). *< P=0.05, **< P=0.001, ***< P=0.0001.

To address the involvement of autophagy induction in host cell death, we used ATG5 or ATG7-deficient mice in intestinal epithelial cells. Specifically, we used the Villin-cre and flox system proteins (Villin-cre xATG5^flox/flox^ =ATG5^∆IEC^ or Villin-cre xATG7^flox/flox^ =ATG7^∆IEC^), which are crucial for the autophagy pathway [8]. A previous study reported abnormalities of Paneth cells in the crypt when autophagy was blocked in intestinal epithelial cells [19]. When ATG7^∆IEC^ mice were orally infected with M90T, cell death and epithelial shedding in the ileum were significantly increased compared with findings in ATG7 intact wild-type (ATG7^fl/fl^) mice at recovery phase 24 h (Figure 5A). In addition, goblet cell hypertrophy and mucus secretion was prominently enhanced by 1 h post infection (Figure S9A). To test whether enhanced cell death and tissue destruction was ascribable to increased bacterial invasion, tissue CFU were examined. M90T invasion inside ileum and MLNs was greatly increased while bacterial shedding in the feces was significantly decreased (Figure 5B-C). Most importantly, the ATG7^∆IEC^ ileum showed inflammation as evidenced by leukocyte infiltration and crypt loss 24 h after oral infection (Figure 5D). The inflammatory cytokines, TNF-α and IFN-γ from the ATG7^∆IEC^ ileum, were significantly elevated compared to those of ATG7^fl/fl^ mice, which showed no signs of inflammation after oral *Shigella* infection (Figure 5E). In addition, CD11b^+^ monocyte-like cells infiltrated into the inflammatory sites of the ATG7^∆IEC^ ileum (Figure 5F). Consistent with this, MCP-1 secretion for monocyte chemoattractant was increased in ATG7^∆IEC^ mice, although CXCL1/KC secretion for neutrophil chemoattractant showed minimal changes (Figure S9B). ATG5^∆IEC^ mice, which are deficient for other autophagy-related components, also showed after oral *Shigella* infection responses similar to those of ATG7^∆IEC^ mice, demonstrating enhanced shedding of epithelium, cell death in the crypt regions, and inflammation in the ileum (Figure S10). The enhanced tissue damage and inflammation in the absence of autophagy was also transient and recovered within a few days (data not shown). Collectively, these results demonstrate that autophagy in the intestinal epithelium confers another level of protection against invasive bacteria and helps to repress pathologic inflammation. 

## Discussion

*Shigella* *spp.* enter the colonic epithelium via M cell translocation, invade resident macrophages and dendritic cells, and multiply within the intestinal epithelium, ultimately leading to severe inflammatory colitis that is called bacillary dysentery (or shigellosis) [1,20]. Although this infection journey must also occur in humans, it has been generally accepted that nothing happens to adult mice. We and other groups have tried to establish an animal model for *Shigella* infection using neonate mice [6,7] or young guinea pigs [21]. Here we propose there is clear evidence of aggressive intrinsic host responses in adult mice. After sacrifice of infected and bystander host cells, there is rapid renewal of epithelium. 

Epithelial cell exfoliation is an intrinsic host defense to bacterial infections in which damaged host cells and colonized pathogens are quickly expelled from the epithelial lining [22]. Infected epithelial cells have an alarm system to alert uninfected neighboring cells by transferring danger signals via the gap junction [23]. This mechanism enables epithelial cells to detach as a group from basement membrane and to dispose of suspected host cells. Therefore, enteric bacteria have evolved various mechanisms to manipulate host cells to enable colonization and invasion to progress to pathogenesis. For natural epithelial cell shedding, cells on villi tips (about 3% of cells) are exfoliated in human and mouse intestines [22]. Epithelial turnover accelerates when tissue damage is caused by bacterial infection. Some bacterial proteins are involved in impairment of host epithelial barrier function. In this regard, shigellae deliver IpaB via the T3SS, resulting in cell-cycle arrest at the G2/M phase and enabling the pathogen to inhibit epithelial renewal and promote bacterial colonization [24]. *Shigella* *spp.* also prevent intestinal epithelial cell detachment by delivering OspE, which targets a host integrin-linked kinase and reinforces epithelial adhesion to the basal lamina [25]. Our findings show that intrinsic host defense mechanisms (i.e., acute cell death) begin by overtaking *Shigella* intestinal epithelial cell manipulation at the early stage of infection. 

Pattern recognition innate receptor NOD1 delivers positive signals to surviving host cells in nonmyeloid cells [26]. Nonmyeloid cells show necrotic death whereas macrophages undergo pyroptosis that is dependent on caspase-1 signaling. To favor host cell survival and initiate the proinflammatory cascade, NF-κB activation is considered critical. A recent study showed constitutive activation of NF-κB by transgenic IKK-β expression in intestinal epithelial cells does not result in tissue damage because they require additional activation of p38 and mitogen-activated protein kinases to induce destructive inflammation [27]. Consistent with previous reports, our results also show that epithelial cells die by necrosis whereas LP cells undergo apoptosis when exposed to the lumen environment. However, these cell deaths were not accompanied by intestinal inflammation since shigellae have an active immune suppression mechanism via the deamidation of UBC13 by OspI [28].

Autophagy mediates the well-organized intracellular disposal system of abnormal cell organelles/molecules or micro-invaders [8]. The cells deficient in Atg5, one of the autophagic mechanisms, fail to control GAS [10]. Thus, the autophagic process can act as an innate defense system against invading pathogens. *Shigella* *spp.* also could be a target of autophagy. After shigellae invade epithelial cells, septin rings assemble at sites of VirG-induced actin polymerization and form cages that arrest the bacterium, which, in turn, help the pathogen to be targeted by autophagy [29]. Phagocytic vacuolar membrane remnants, which are host membranes after rupture by invading intracellular bacteria, contribute to a signaling node of autophagy [30]. However, wild-type *Shigella* *spp.* can evade autophagy by delivering IcsB, which competitively binds to VirG to block Atg5 binding. Tectonin domain-containing protein (Tecpr1) also interacts with the Atg12-Atg5-Atg16L1 complex via binding to Atg5, which promotes selective autophagy via the WIPI-2-Tecpr1-Atg5 pathway in targeting bacteria [31]. Nod1 and Nod2, which enable host cells to recognize *Shigella* organisms, also can act as initiation modules of autophagy by recruiting Atg16L1 to the plasma membrane at the site of bacterial entry [32]. When autophagy was blocked in the epithelial cells, the host intestine became more vulnerable to death stimuli by *Shigella* infection.

Selective autophagy can tackle microbes and attenuate endotoxin-induced inflammatory responses in intestinal epithelium resulting in the maintenance of intestinal homeostasis [33]. Atg16L1-deficient macrophages produce high amounts of the inflammatory cytokines IL-1β and IL-18 [34]. Mice lacking Atg16L1 in hematopoietic cells are highly susceptible to dextran sulfate sodium (DSS)-induced acute colitis. Others have found that Paneth cell abnormalities in Atg16L1^HM^ mice are triggered by infection with a murine norovirus strain [35,36]. The response to injury induced by the toxic substance DSS was aggravated in these mice. Autophagic proteins were also found to regulate NALP3-dependent inflammation by preserving mitochondrial integrity [37]. In the mouse small intestine, the common cellular target of Atg16L1, Atg5, and Atg7 is the Paneth cell, a specialized epithelial cell whose main function is the delivery of antimicrobial factors into the intestinal lumen by production and secretion of its characteristic cytoplasmic granules [19]. Autophagy-deficient Paneth cells exhibited a striking loss of function in this granule exocytosis pathway. After *Shigella* infection, canonical autophagy induction, but not xenophagy, which is microbe-selective, was evident in the crypt as well as epithelium although non-infected cells also could induce autophagy at low levels. In the present study *Shigella* infection finally induced inflammation when autophagy was blocked in the epithelial cells. Therefore, our data suggest that increased autophagy pathway in the intestine could modulate inflammatory response to maintain intestinal homeostasis.

Here we propose autophagy as the concept of a general intrinsic mechanism to repel massive introduction of entero-pathogenic bacteria. During the acute enteric infection process, the intestinal environment undergoes sudden changes ranging from cell death and tissue destruction to renewal of normal structures of the host intestine. However, this process does not link destructive inflammation due to the rapid autophagy response, although there are losses of tissue integrity and exposure of naive LP to the intestinal lumen. Autophagy flow induced against bacterial infection might relieve cellular stress to induce inflammation. These findings may help in the understanding of the crosstalk between a host and microbes.

## Supporting Information

Figure S1 **Evaluation of pathological score from H&E histology of terminal ileum tissues following oral M90T or other entero-pathogenic bacterial infection.** (A) Severity grade and screening criteria of host cell death and tissue damage for pathological evaluation following oral M90T infection. (B) Criteria dissection for pathological score of terminal ileum following oral M90T infection shown in Figure 2B.
(JPG)

Figure S2 ***Shigella* M90T infection found in terminal ileum.** (A) PP and LP of duodenum, jejunum, and ileum 1 hour after oral M90T infection. (B) Body weight change and H&E histology following oral infection with low doses of M90T. (C) H&E histology of ileum following low dose of oral M90T infection. (D-E) Body weight changes following oral gavage with 5 x 10^9^ virulent *Shigella flexneri* 5a (M90T), avirulent *S. flexneri* 5a (BS176), and T3SS deleted mutants (M90T∆IpaD or M90T∆IpaB4). Data are representative of three independent experiments. *< P=0.05, ***< P=0.0001. (F) H&E histology of ileum following oral M90T∆IpaB4 infection.
(JPG)

Figure S3 **Gene expression profile analysis following oral M90T infection by cDNA microarray.** mRNA was extracted from terminal ileum tissues 1 hour after oral M90T infection. (A) Analysis of gene expression in naive vs. M90T-infected ileum. (B) Selected genes from gene chip analysis were confirmed by real-time PCR. (C) Selected genes were analyzed for transcriptional expression as the ratio of M90T (1h) / naïve ileum from gene chip data.
(PDF)

Figure S4 **SEM images of PP in the terminal ileum following oral M90T infection.**
(JPG)

Figure S5 **SEM images of intestinal villi in the terminal ileum 1 hour following oral M90T infection.** Lamina propria (LP) and EC in the epithelium (E).
(JPG)

Figure S6 **Entero-pathogenic bacterial infection can induce host cell death and tissue injuries regardless of presence of Shiga toxin.** Mice were orally infected with 5 x 10^9^ bacteria before analysis of terminal ileum. H&E histology and pathological score of terminal ileum tissue following *Shigella flexneri 2a* (YSH6000) (A) and *Shigella dysenteriae 1* (B). Not significant (ns), *< P=0.05, ***< P=0.0001. (C) Mice were orally administered with lysates of 5 x 10^9^ M90T strain. H&E histology of terminal ileum tissue at 1 hour.
(JPG)

Figure S7 **Alteration of Paneth cells in the crypt following oral M90T infection.** Periodic acid-Schiff (PAS) (A) and lysozyme (B) staining of the terminal ileum.
(JPG)

Figure S8 **Cell death following oral M90T infection.** Isolated intestinal EC were stained with anti-Annexin V and PI to determine cell death following oral M90T infection.
(JPG)

Figure S9 **Blockade of autophagy in the epithelium of ATG7^∆IEC^ mice induced hypertrophy of goblet cells and enhanced mucus secretion following oral M90T infection.** ATG7^fl/fl^ or ATG7^∆IEC^ mice were infected with oral M90T and terminal ilea were analyzed according to time course. (A) H&E and PAS staining of terminal ileum at 1 hour after infection. Representative images and pathologic scores for enhanced mucus secretion. (B) Levels of MCP-1, CXCL1/KC, and IL-6 from ileum tissue homogenates of ATG7^fl/fl^ or ATG7^∆IEC^ mice at 24 hours after infection. Not significant (ns), *< P=0.05.
(JPG)

Figure S10 **Blockade of autophagy in the epithelium of ATG5^∆IEC^ mice induced increased cell death and inflammation following oral M90T infection.** ATG5^fl/fl^ or ATG5^∆IEC^ mice were infected with oral M90T and then terminal ilea were analyzed by H&E staining according to time course. Representative images and pathologic scores for cell death and shedding (A), for crypt cell death (B), and for inflammation (C). Not significant (ns), *< P=0.05.
(JPG)

Table S1 **List of specific primer sets used for real time-PCR.**
(PDF)

Table S2 **List of the gene in the biological process whose expression levels were different by more than two-fold between naïve and M90T-infected mice.**
(PDF)
