# Supplementary figures and images for: Autophagy Controls an Intrinsic Host Defense to Bacteria by Promoting Epithelial Cell Survival: A Murine Model
Source: PLoS One. 2013 Nov 19;8(11):e81095. doi: 10.1371/journal.pone.0081095 (PMC3834267; doi:10.1371/journal.pone.0081095)

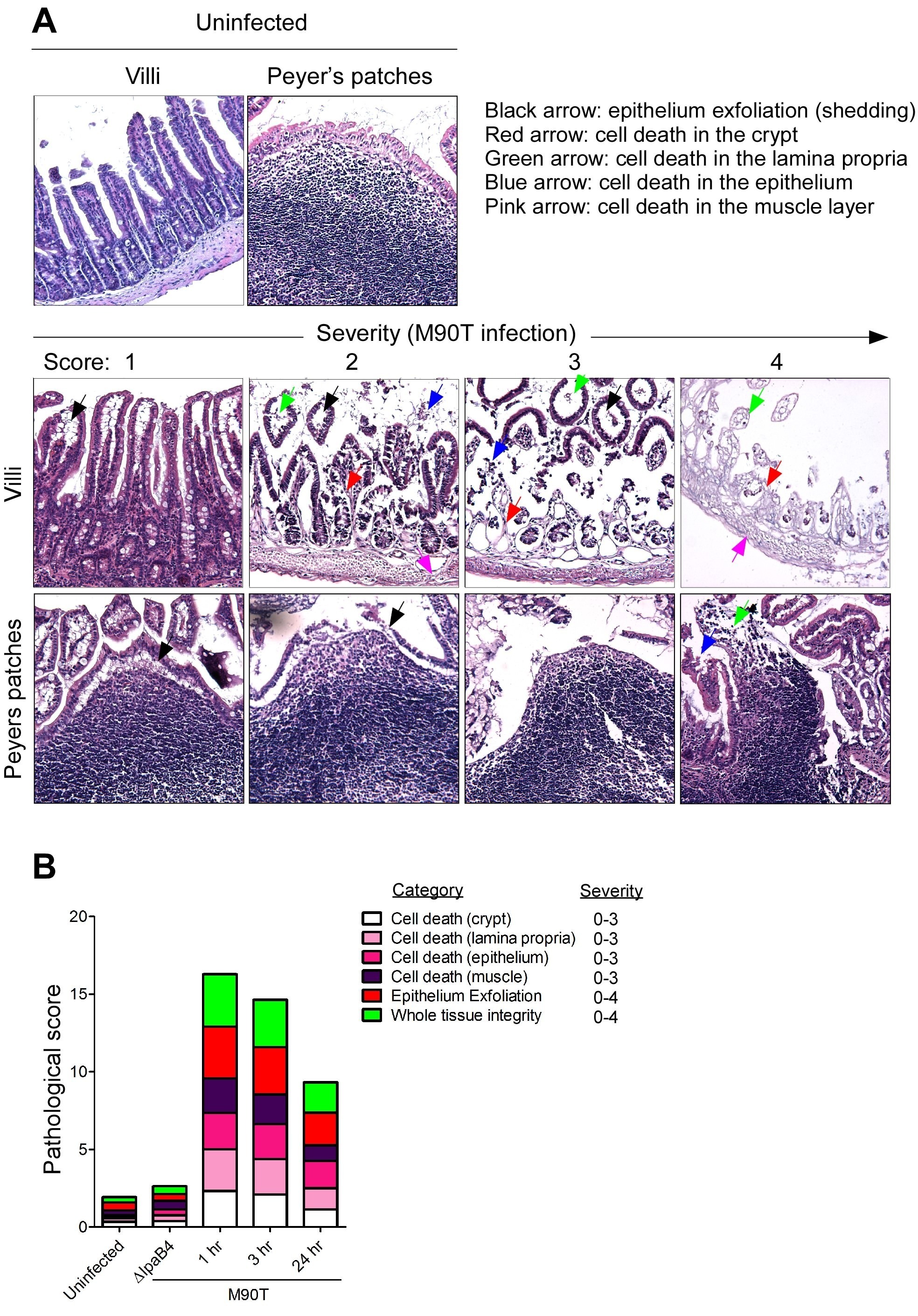

Supplement: Figure S1 — Evaluation of pathological score from H&E histology of terminal ileum tissues following oral M90T or other entero-pathogenic bacterial infection. (A) Severity grade and screening criteria of host cell death and tissue damage for pathological evaluation following oral M90T infection. (B) Criteria dissection for pathological score of terminal ileum following oral M90T infection shown in Figure 2B. (JPG) [file pone.0081095.s001.jpg]

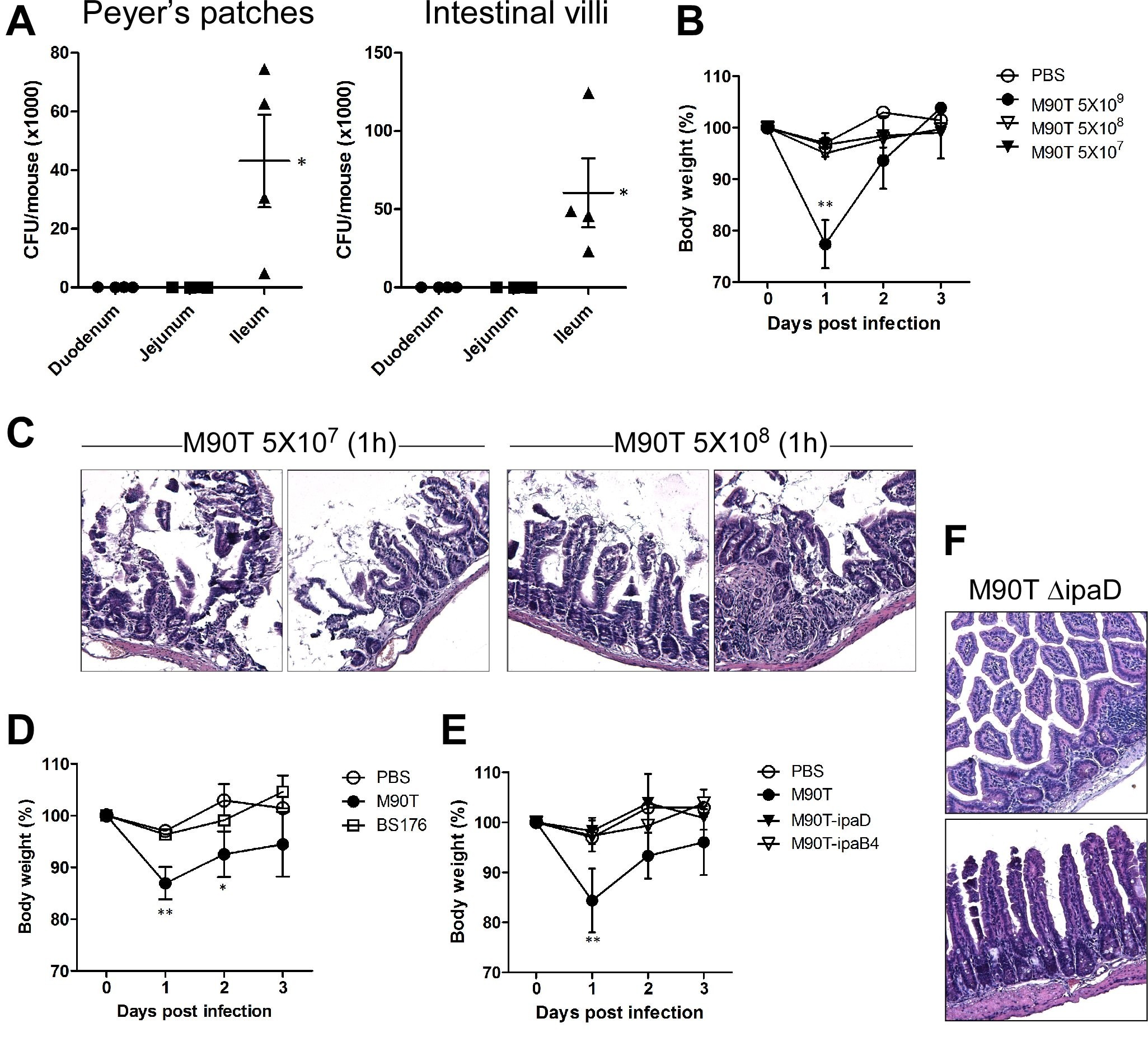

Supplement: Figure S2 — Shigella M90T infection found in terminal ileum. (A) PP and LP of duodenum, jejunum, and ileum 1 hour after oral M90T infection. (B) Body weight change and H&E histology following oral infection with low doses of M90T. (C) H&E histology of ileum following low dose of oral M90T infection. (D-E) Body weight changes following oral gavage with 5 x 10^9^ virulent Shigella flexneri 5a (M90T), avirulent S. flexneri 5a (BS176), and T3SS deleted mutants (M90T∆IpaD or M90T∆IpaB4). Data are representative of three independent experiments. *< P=0.05, ***< P=0.0001. (F) H&E histology of ileum following oral M90T∆IpaB4 infection. (JPG) [file pone.0081095.s002.jpg]

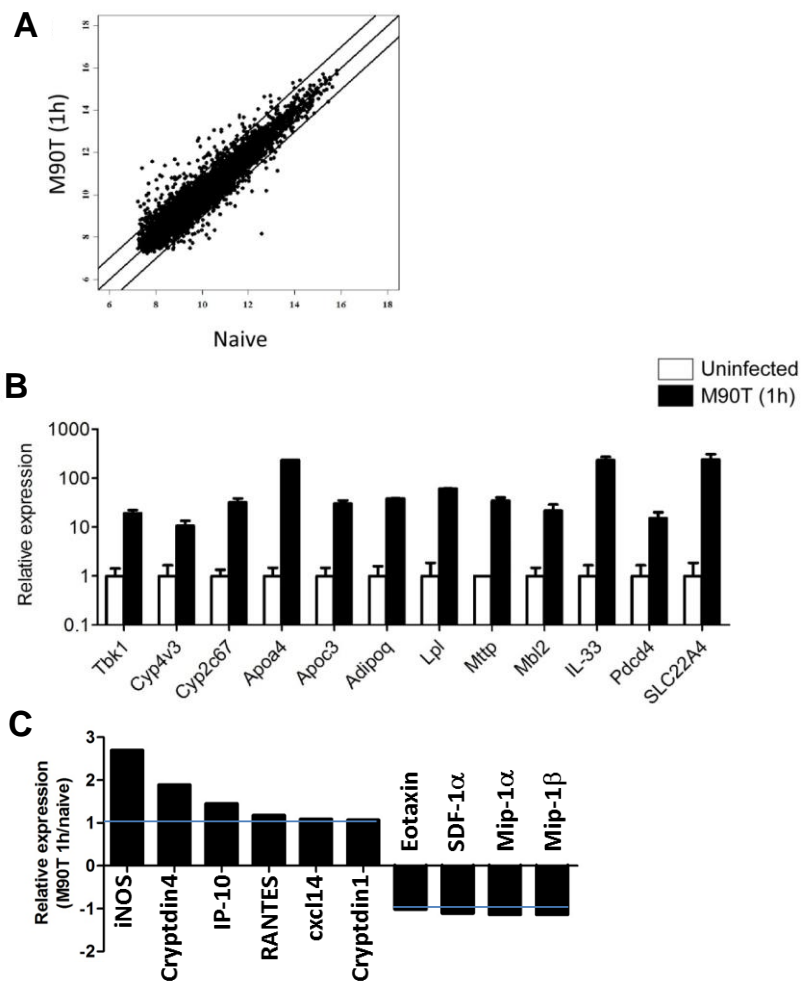

Supplement: Figure S3 — Gene expression profile analysis following oral M90T infection by cDNA microarray. mRNA was extracted from terminal ileum tissues 1 hour after oral M90T infection. (A) Analysis of gene expression in naive vs. M90T-infected ileum. (B) Selected genes from gene chip analysis were confirmed by real-time PCR. (C) Selected genes were analyzed for transcriptional expression as the ratio of M90T (1h) / naïve ileum from gene chip data. (PDF) [file pone.0081095.s003.pdf]

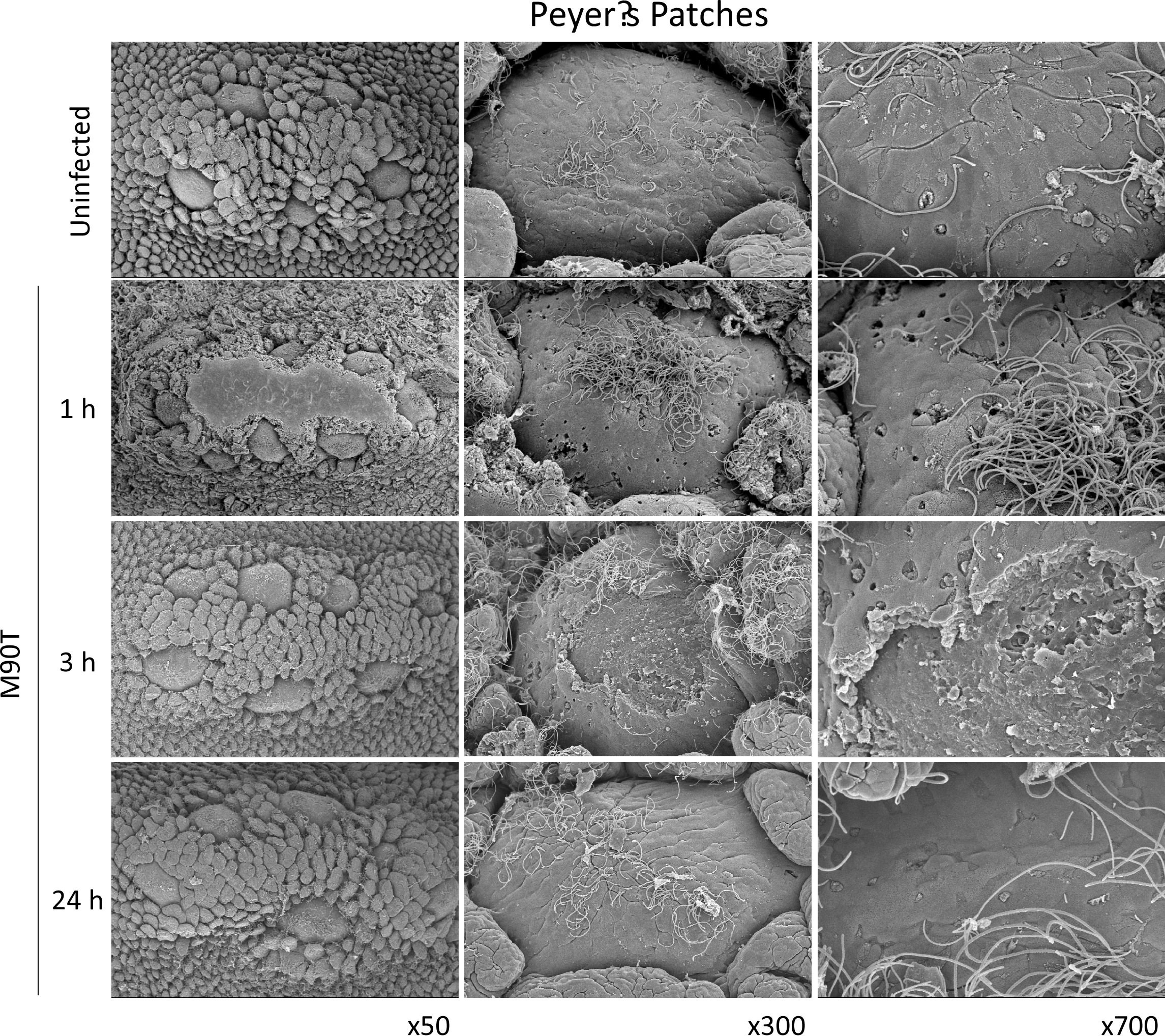

Supplement: Figure S4 — SEM images of PP in the terminal ileum following oral M90T infection. (JPG) [file pone.0081095.s004.jpg]

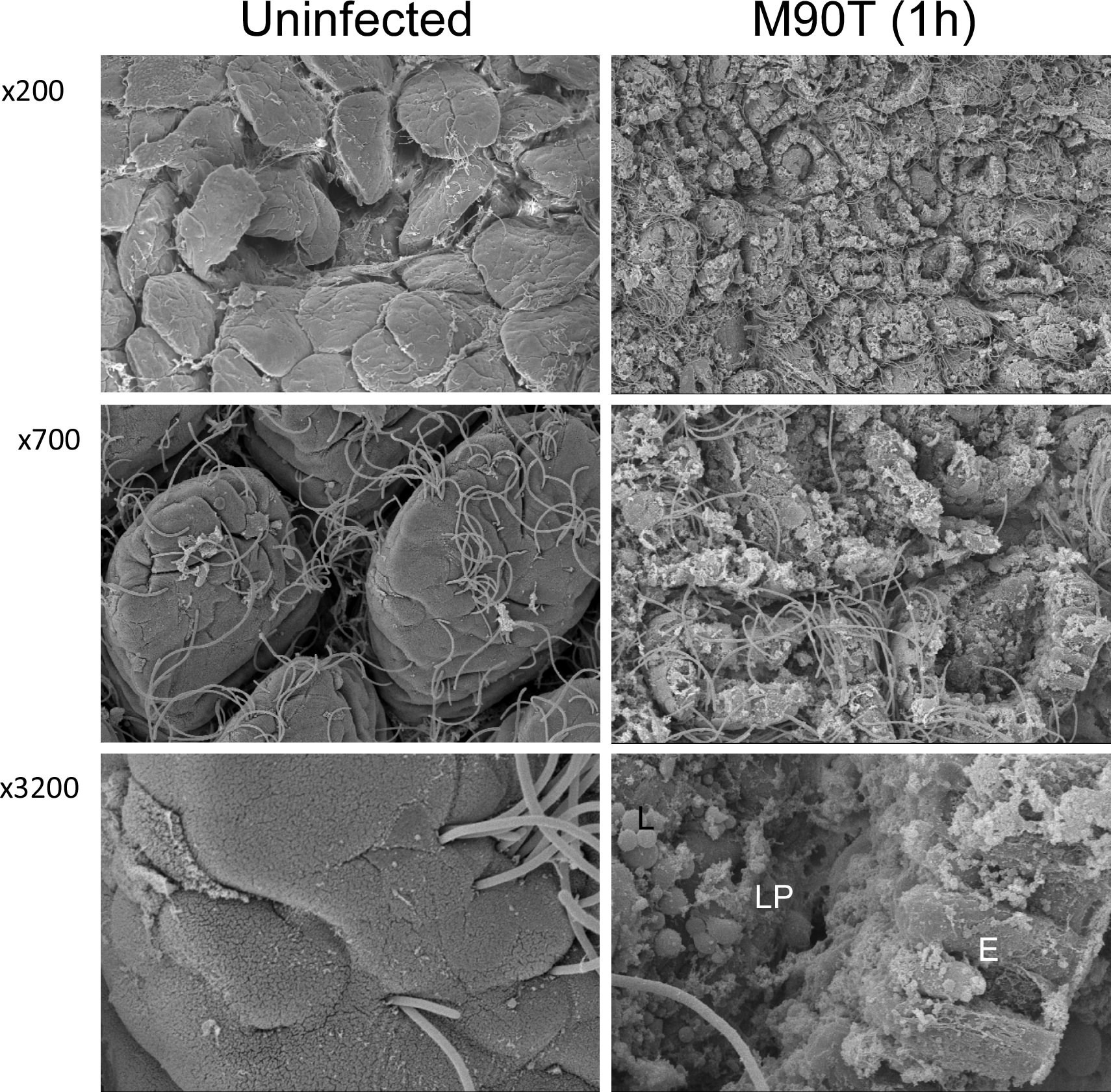

Supplement: Figure S5 — SEM images of intestinal villi in the terminal ileum 1 hour following oral M90T infection. Lamina propria (LP) and EC in the epithelium (E). (JPG) [file pone.0081095.s005.jpg]

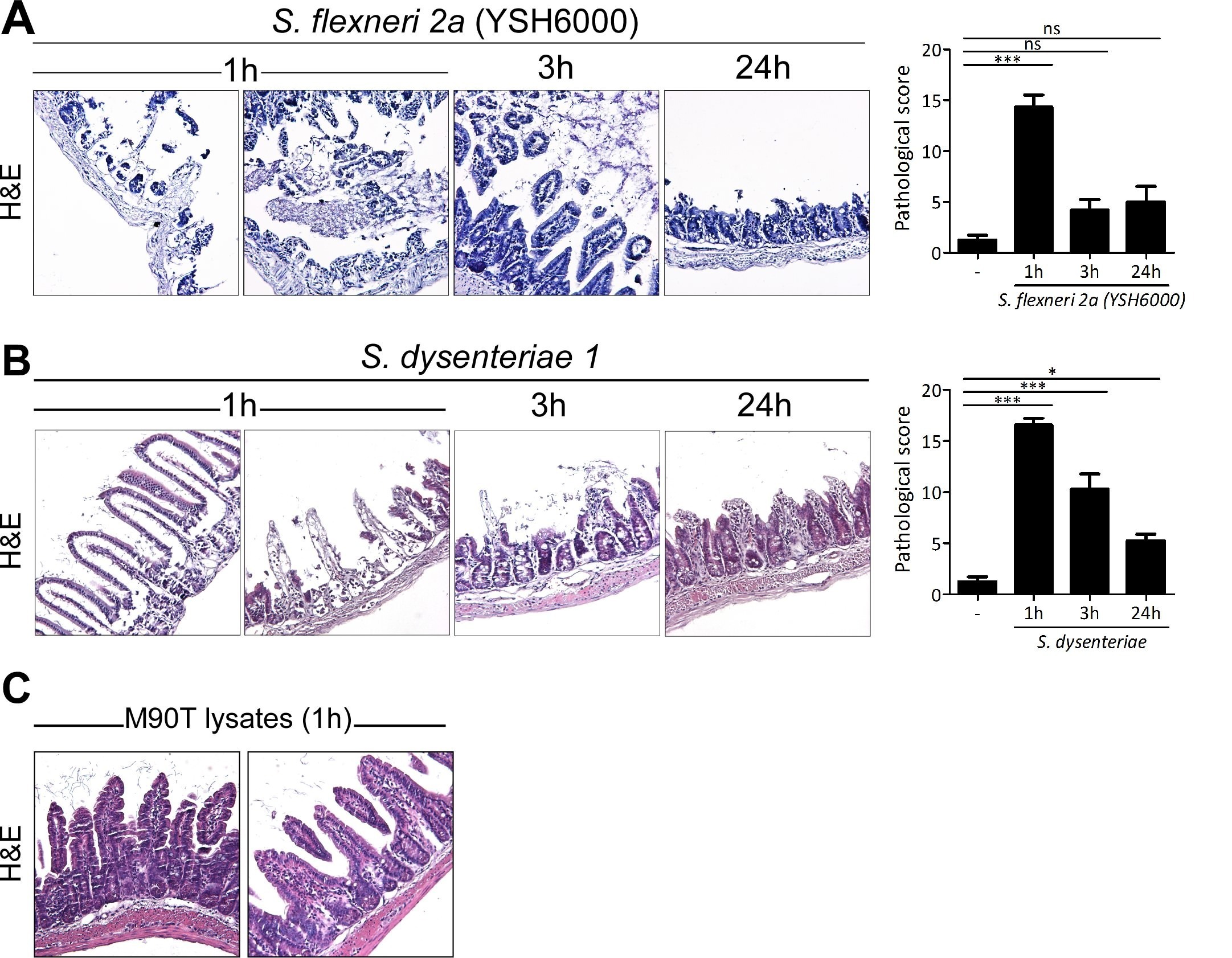

Supplement: Figure S6 — Entero-pathogenic bacterial infection can induce host cell death and tissue injuries regardless of presence of Shiga toxin. Mice were orally infected with 5 x 10^9^ bacteria before analysis of terminal ileum. H&E histology and pathological score of terminal ileum tissue following Shigella flexneri 2a (YSH6000) (A) and Shigella dysenteriae 1 (B). Not significant (ns), *< P=0.05, ***< P=0.0001. (C) Mice were orally administered with lysates of 5 x 10^9^ M90T strain. H&E histology of terminal ileum tissue at 1 hour. (JPG) [file pone.0081095.s006.jpg]

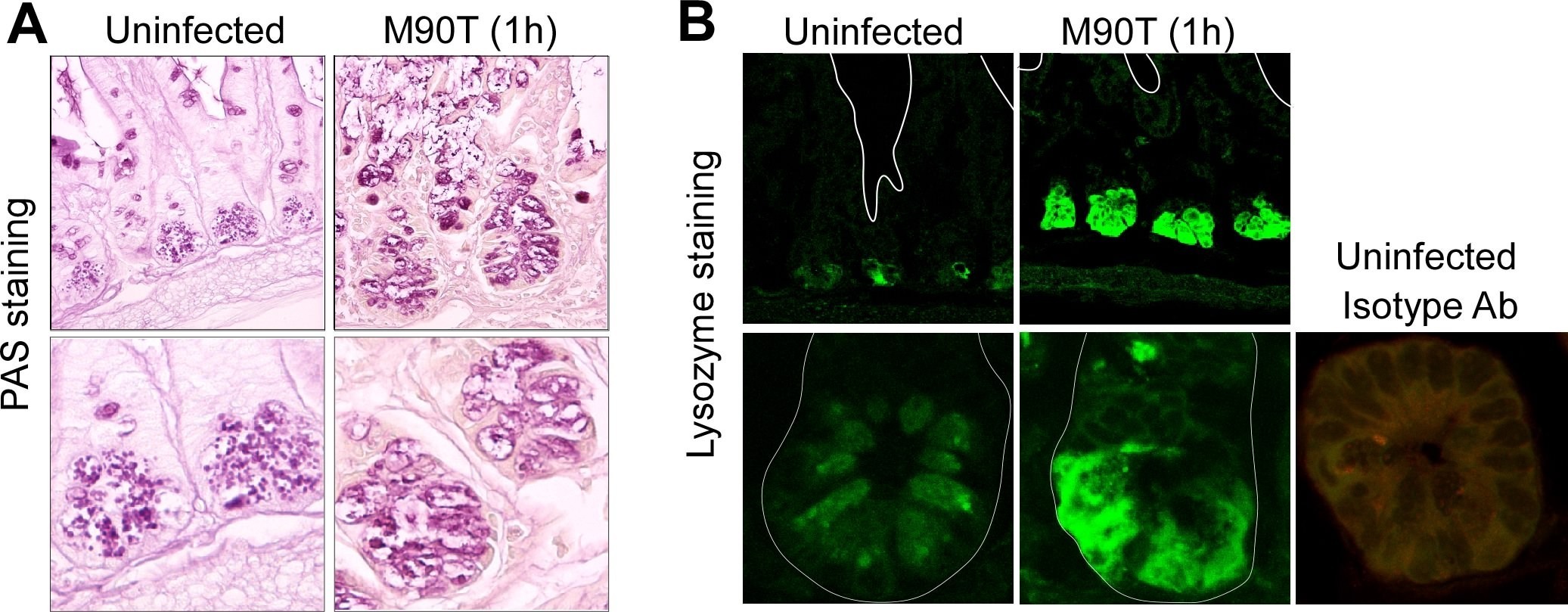

Supplement: Figure S7 — Alteration of Paneth cells in the crypt following oral M90T infection. Periodic acid-Schiff (PAS) (A) and lysozyme (B) staining of the terminal ileum. (JPG) [file pone.0081095.s007.jpg]

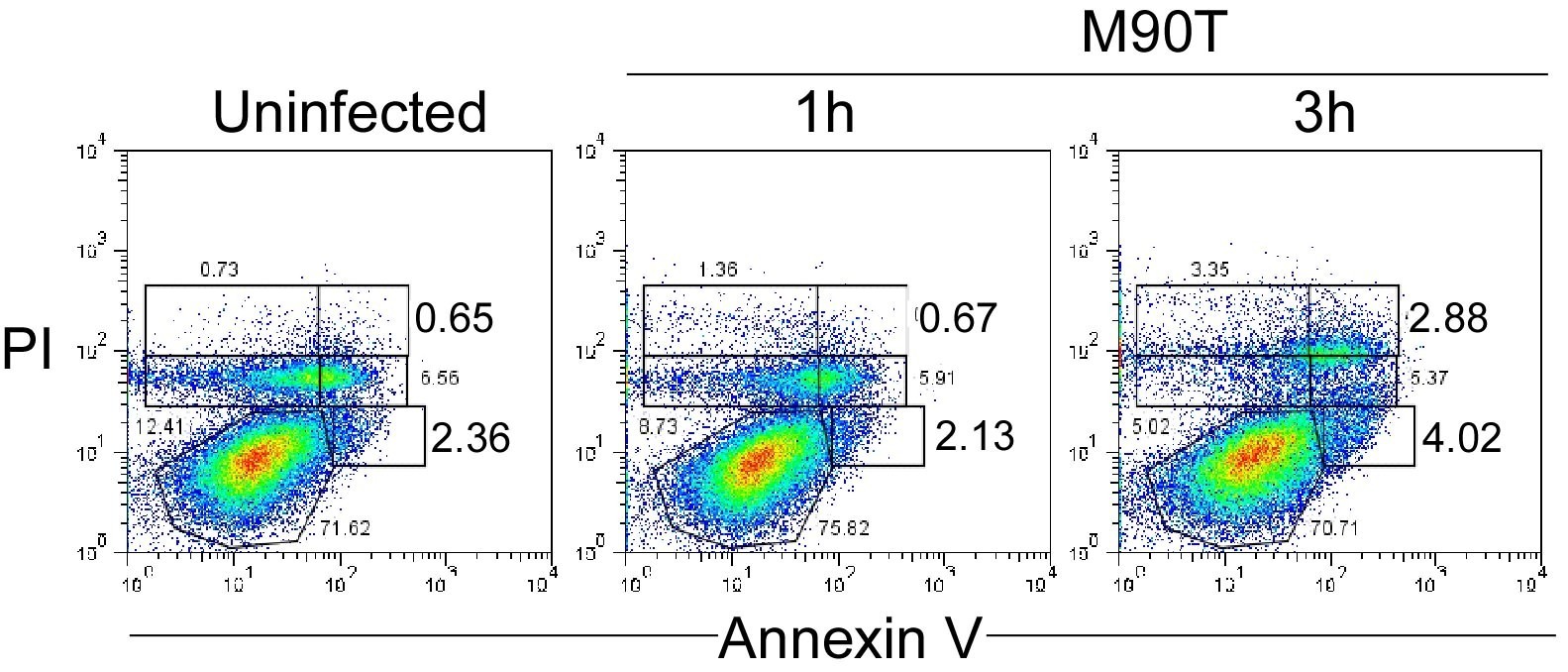

Supplement: Figure S8 — Cell death following oral M90T infection. Isolated intestinal EC were stained with anti-Annexin V and PI to determine cell death following oral M90T infection. (JPG) [file pone.0081095.s008.jpg]

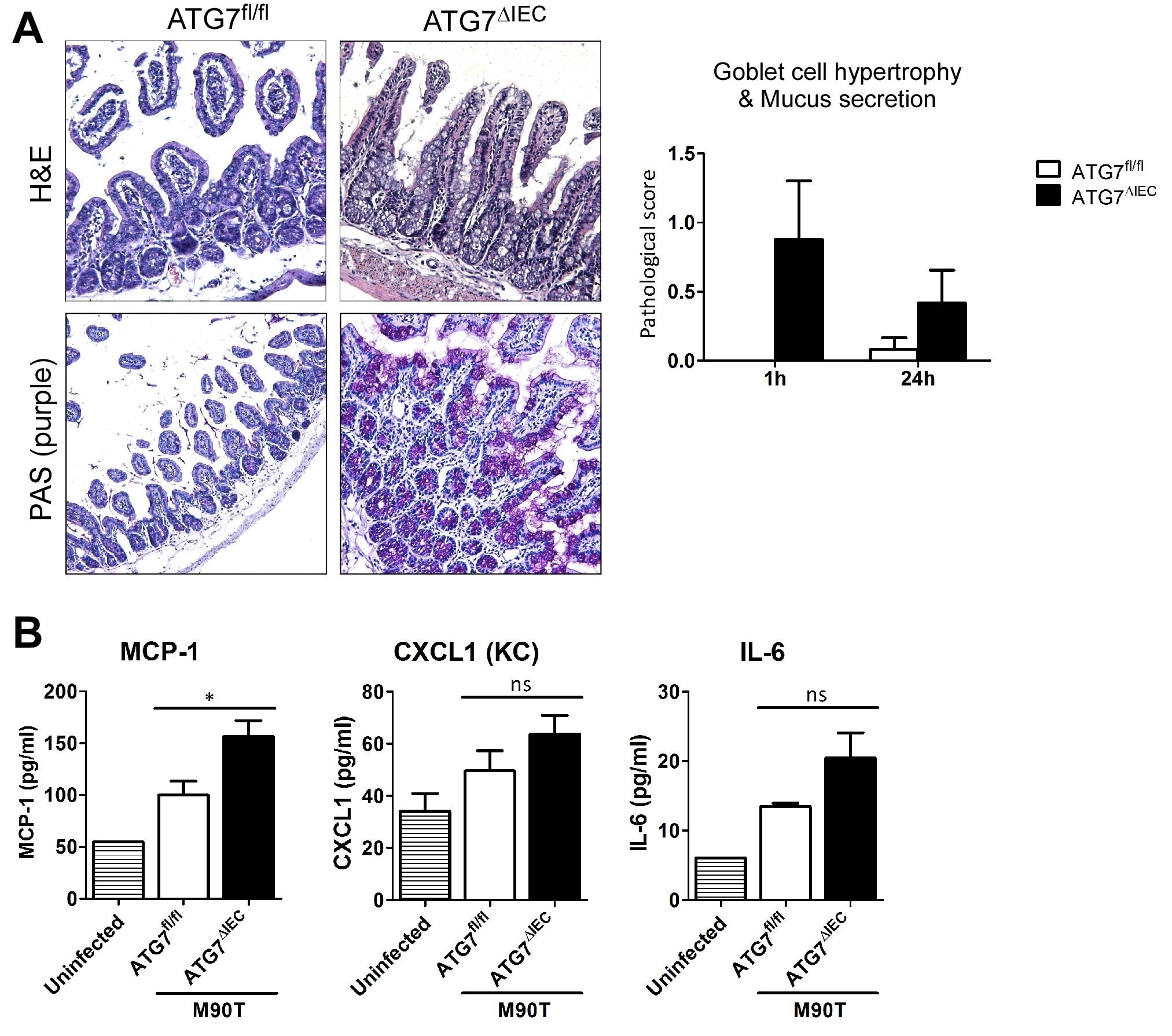

Supplement: Figure S9 — Blockade of autophagy in the epithelium of ATG7∆IEC mice induced hypertrophy of goblet cells and enhanced mucus secretion following oral M90T infection. ATG7fl/fl or ATG7∆IEC mice were infected with oral M90T and terminal ilea were analyzed according to time course. (A) H&E and PAS staining of terminal ileum at 1 hour after infection. Representative images and pathologic scores for enhanced mucus secretion. (B) Levels of MCP-1, CXCL1/KC, and IL-6 from ileum tissue homogenates of ATG7fl/fl or ATG7∆IEC mice at 24 hours after infection. Not significant (ns), *< P=0.05. (JPG) [file pone.0081095.s009.jpg]

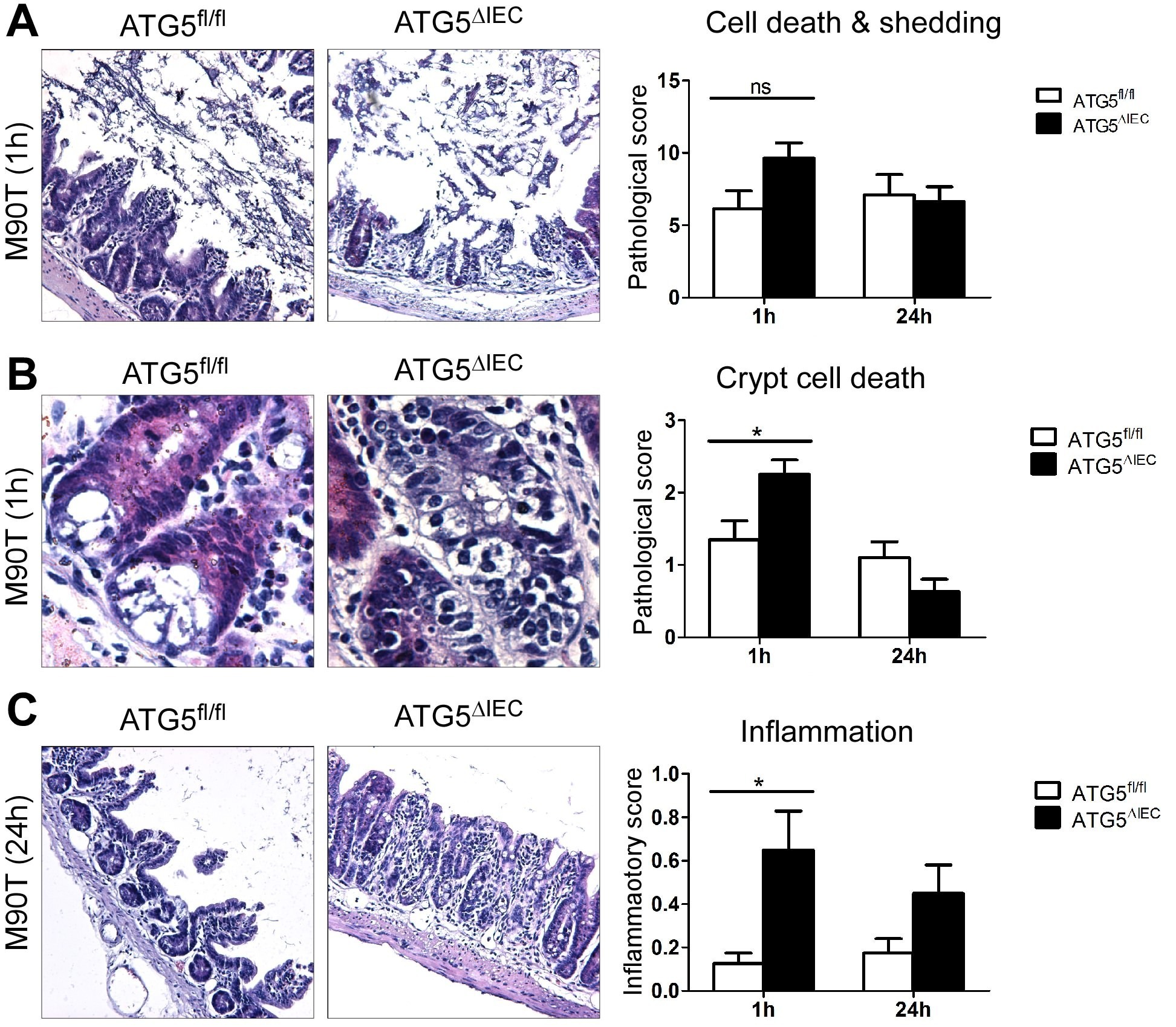

Supplement: Figure S10 — Blockade of autophagy in the epithelium of ATG5∆IEC mice induced increased cell death and inflammation following oral M90T infection. ATG5fl/fl or ATG5∆IEC mice were infected with oral M90T and then terminal ilea were analyzed by H&E staining according to time course. Representative images and pathologic scores for cell death and shedding (A), for crypt cell death (B), and for inflammation (C). Not significant (ns), *< P=0.05. (JPG) [file pone.0081095.s010.jpg]
