# Supplementary material for: Autophagy Controls an Intrinsic Host Defense to Bacteria by Promoting Epithelial Cell Survival: A Murine Model
Source: PLoS One. 2013 Nov 19;8(11):e81095. doi: 10.1371/journal.pone.0081095 (PMC3834267; doi:10.1371/journal.pone.0081095)
Supplement: Table S1 — List of specific primer sets used for real time-PCR. (PDF) [file pone.0081095.s011.pdf]

Table S1. List of specific primer sets used for real time-PCR

Target gene symbol	Product size (bp)	Forward sequence	Reverse sequence
SLC22A4	232	CCTGTTCTGTGTTCCCCTGT	GGTTATGGTGGCAATGTTCC
Il-33	207	TTGGCTTACGATGTTGTGGA	ACTGTGGTGCCTGCTCTTCT
Lpl	167	GGGCTCTGCCTGAGTTGTAG	CCATCCTCAGTCCCAGAAAA
Mttp	162	CACTCAGGCAATTCGAGACA	TATCGCTTTCTGGCTGAGGT
Mbl2	229	ATTGCCTACTTGGGCATCAC	GAGGGCTGAGAAACAAGCAC
Adipoq	192	GTTGCAAGCTCTCCTGTTCC	TCTCCAGGAGTGCCATCTCT
Pdcd4	235	GGAGGGACAGAAGAGGAACC	CTCAAAGCACAGCATTTCCA
Tbk1	208	GAGTACCTGCATCCGGACAT	ATATTGCACCAGACGGCTTC
Apoc3	158	CTAACCCTGAGGACCAACCA	GGAGGGGTGAAGACATGAGA
Apoa4	173	CCTTTATGAGCACCCTGGAA	AGGTGTCTGCTGCTGTGATG
Cyp4v3	227	CCCAACAACGCAGAATTTTT	CATTTGCTCCCCGTACTTGT
Cyp2c67	168	GCAGGAAAACGGATTTGTGT	CATCTGGAAATTGGGAGGAA
DNase1	157	ACTCAATCGGGACAAACCTG	ATTTCCACAGGGTTCACAGC
Maob	160	TGATCTCTCGTGTGCCTTTG	GCATAGGTGCCATCTGGTTT

Supplementary Table 2. List of the gene in the biological process whose expression levels were different by more than two-fold between naïve and M90T-infected mice.

Target ID	Symbol	Naive	M90T (1h)	Fold change (M90T/naive)
**Cell communication and signal transduction**				
ILMN_2597769	Igf2	270.31	1572.23	**6.41**
ILMN_2870672	Fbln1	528.59	2753.71	**5.63**
ILMN_2813859	Rbp2	560.10	1771.88	**3.57**
ILMN_1241535	Tbk1	2693.14	5458.57	**2.02**
ILMN_1251000	Tac1	1291.43	361.91	**-3.18**
ILMN_2652500	Lrg1	1315.54	235.86	**-5.10**
**Metabolism**				
ILMN_1251504	Cyp4v3	2394.89	9792.75	**4.26**
ILMN_3074610	Cyp2c67	486.28	1378.84	**3.18**
ILMN_2834123	Apoa4	977.74	6471.89	**7.03**
ILMN_2651539	Apoc3	1898.27	8090.53	**4.47**
ILMN_2980044	Asah2	1531.31	8591.21	**5.80**
ILMN_2990661	Pnliprp2	1028.51	4836.58	**5.12**
ILMN_1246265	Clps	1900.15	8603.42	**4.84**
ILMN_2871249	Rdh7	212.29	1008.69	**5.17**
ILMN_2738082	Adipoq	165.14	482.38	**2.68**
ILMN_2692723	Lpl	372.78	796.91	**2.17**
ILMN_2628594	Mttp	950.98	1969.08	**2.04**
**Immunity**				
ILMN_2835423	Cfd	170.93	2352.28	**14.48**
ILMN_1258500	Mbl2	162.16	423.69	**2.79**
ILMN_1259747	IL-33	218.23	455.13	**2.17**
ILMN_1251000	Tac1	1291.43	361.91	**-3.18**
ILMN_2826869	Saa1	775.92	221.83	**-3.09**
**Apoptotic processes**				
ILMN_2734251	Dnase1	240.02	2985.49	**13.20**
ILMN_2719069	Maob	357.64	2865.63	**7.92**
ILMN_1222679	Cidec	272.34	580.79	**2.38**
ILMN_2898878	Pdcd4	285.14	511.03	**2.02**
ILMN_1232884	Sphk1	269.60	807.10	**3.35**
ILMN_2698046	Stat3	589.72	1216.34	**2.29**
**Transport**				
ILMN_2598877	Slc7a8	303.61	1857.48	**6.79**
ILMN_2861259	Slc2a2	281.58	1190.28	**4.65**
ILMN_1223035	Slc22a4	323.36	650.67	**2.28**
ILMN_1251109	Slc25a45	478.49	3189.66	**7.30**
ILMN_2943040	Slc15a1	189.61	574.43	**3.27**
**Cell structure**				
ILMN_1249976	Cfl2	325.79	1800.40	**6.06**
ILMN_2617433	Actb	492.37	1604.52	**3.71**
ILMN_2738825	Acta1	6217.79	270.19	**-21.53**
