# Supplementary material for: Autophagy Controls an Intrinsic Host Defense to Bacteria by Promoting Epithelial Cell Survival: A Murine Model
Source: PLoS One. 2013 Nov 19;8(11):e81095. doi: 10.1371/journal.pone.0081095 (PMC3834267; doi:10.1371/journal.pone.0081095)
Supplement: Table S2 — List of the gene in the biological process whose expression levels were different by more than two-fold between naïve and M90T-infected mice. (PDF) [file pone.0081095.s012.pdf]

Table S2. List of the gene in the biological process whose expression levels were different by more than two-fold between naïve and M90T-infected mice.

Target ID	Symbol	Naive	M90T (1h)	Fold change (M90T/naive)
**Cell communication and signal transduction**				
ILMN_2597769	Igf2	270.31	1572.23	**6.41**
ILMN_2870672	Fbln1	528.59	2753.71	**5.63**
ILMN_2813859	Rbp2	560.10	1771.88	**3.57**
ILMN_1241535	Tbk1	2693.14	5458.57	**2.02**
ILMN_1251000	Tac1	1291.43	361.91	**-3.18**
ILMN_2652500	Lrg1	1315.54	235.86	**-5.10**
**Metabolism**				
ILMN_1251504	Cyp4v3	2394.89	9792.75	**4.26**
ILMN_3074610	Cyp2c67	486.28	1378.84	**3.18**
ILMN_2834123	Apoa4	977.74	6471.89	**7.03**
ILMN_2651539	Apoc3	1898.27	8090.53	**4.47**
ILMN_2980044	Asah2	1531.31	8591.21	**5.80**
ILMN_2990661	Pnliprp2	1028.51	4836.58	**5.12**
ILMN_1246265	Clps	1900.15	8603.42	**4.84**
ILMN_2871249	Rdh7	212.29	1008.69	**5.17**
ILMN_2738082	Adipoq	165.14	482.38	**2.68**
ILMN_2692723	Lpl	372.78	796.91	**2.17**
ILMN_2628594	Mttp	950.98	1969.08	**2.04**
**Immunity**				
ILMN_2835423	Cfd	170.93	2352.28	**14.48**
ILMN_1258500	Mbl2	162.16	423.69	**2.79**
ILMN_1259747	IL-33	218.23	455.13	**2.17**
ILMN_1251000	Tac1	1291.43	361.91	**-3.18**
ILMN_2826869	Saa1	775.92	221.83	**-3.09**
**Apoptotic processes**				
ILMN_2734251	Dnase1	240.02	2985.49	**13.20**
ILMN_2719069	Maob	357.64	2865.63	**7.92**
ILMN_1222679	Cidec	272.34	580.79	**2.38**
ILMN_2898878	Pdcd4	285.14	511.03	**2.02**
ILMN_1232884	Sphk1	269.60	807.10	**3.35**
ILMN_2698046	Stat3	589.72	1216.34	**2.29**
**Transport**				
ILMN_2598877	Slc7a8	303.61	1857.48	**6.79**
ILMN_2861259	Slc2a2	281.58	1190.28	**4.65**
ILMN_1223035	Slc22a4	323.36	650.67	**2.28**
ILMN_1251109	Slc25a45	478.49	3189.66	**7.30**
ILMN_2943040	Slc15a1	189.61	574.43	**3.27**
**Cell structure**				
ILMN_1249976	Cfl2	325.79	1800.40	**6.06**
ILMN_2617433	Actb	492.37	1604.52	**3.71**
ILMN_2738825	Acta1	6217.79	270.19	**-21.53**
